# Machine Learning Prediction of Length of Stay in Adult Spinal Deformity Patients Undergoing Posterior Spine Fusion Surgery

**DOI:** 10.3390/jcm10184074

**Published:** 2021-09-09

**Authors:** Andrew S Zhang, Ashwin Veeramani, Matthew S. Quinn, Daniel Alsoof, Eren O. Kuris, Alan H. Daniels

**Affiliations:** 1 Department of Orthopaedic Surgery, Warren Alpert Medical School of Brown University, Rhode Island Hospital, Providence, RI 02912, USA; andrew_zhang4@brown.edu (A.S.Z.); matthew_quinn@brown.edu (M.S.Q.); daniel_alsoof@brown.edu (D.A.); eokuris@gmail.com (E.O.K.); 2 Division of Applied Mathematics, Brown University, Providence, RI 02912, USA; ashwin_veeramani@brown.edu

**Keywords:** machine learning, length of stay, adult spinal deformity, posterior spine fusion surgery

## Abstract

(1) Background: Length of stay (LOS) is a commonly reported metric used to assess surgical success, patient outcomes, and economic impact. The focus of this study is to use a variety of machine learning algorithms to reliably predict whether a patient undergoing posterior spinal fusion surgery treatment for Adult Spine Deformity (ASD) will experience a prolonged LOS. (2) Methods: Patients undergoing treatment for ASD with posterior spinal fusion surgery were selected from the American College of Surgeon’s NSQIP dataset. Prolonged LOS was defined as a LOS greater than or equal to 9 days. Data was analyzed with the Logistic Regression, Decision Tree, Random Forest, XGBoost, and Gradient Boosting functions in Python with the Sci-Kit learn package. Prediction accuracy and area under the curve (AUC) were calculated. (3) Results: 1281 posterior patients were analyzed. The five algorithms had prediction accuracies between 68% and 83% for posterior cases (AUC: 0.566–0.821). Multivariable regression indicated that increased Work Relative Value Units (RVU), elevated American Society of Anesthesiologists (ASA) class, and longer operating times were linked to longer LOS. (4) Conclusions: Machine learning algorithms can predict if patients will experience an increased LOS following ASD surgery. Therefore, medical resources can be more appropriately allocated towards patients who are at risk of prolonged LOS.

## 1. Introduction

Length of stay (LOS) is a commonly reported metric used to assess surgical success and patient outcomes. However, in the face of rising healthcare costs, LOS is also a measure often targeted to reduce these expenses. Bundled payments have become more prevalent, and therefore, tremendous efforts have been made to safely minimize patient LOS. Procedures such as total shoulder (TSA) and hip arthroplasties (THA) are sometimes performed as outpatient procedures, which are on average 30% less costly than the same procedures that require admission [1].

However, more invasive orthopedic procedures, such as spinal fusion for adult spinal deformity (ASD), cannot inherently make this transition due to patient immobility, intraoperative blood loss, and inadequate perioperative pain control. In such cases, ensuring that patients avoid unnecessary extensions in length of stay is imperative. A study by Boylan et al. conducted on adolescent scoliosis surgery costs indicated that each additional day of hospitalization incurred more than $1100 in insurance costs and close to $5200 in hospitals costs [2]. Furthermore, patients who experience a prolonged LOS can accrue an additional $19,000 in total hospital costs compared to shorter LOS counterparts [3]. Similar trends have been published for spinal fusion surgery for ASD, thereby prompting the need for further research efforts to either reduce LOS or avoid unnecessary extensions [4].

Several studies have identified risk factors for increased LOS after spinal fusion surgery for ASD. Phan et al. found that increased operative time was associated with prolonged LOS for adult spinal deformity surgery [5]. In a multicenter study, Klineberg et al. identified age, heart disease, Charlson Comordity Index scores, number of levels fused, infection, neurologic deficits, and intraoperative complications as risk factors for increased LOS [6]. Another study using Danish hospital data corroborated these findings by showing that increased age and Charlson Comorbidity Index scores lead to increased LOS [7]. Although risk factors are useful in identifying patients predisposed to longer LOS, they cannot deterministically predict whether a given patient will go on to experience a prolonged LOS. However, machine learning algorithms can synthesize these studies and predict whether a given patient will undergo a short or long hospitalization based on several demographic and comorbidity data taken collectively rather than considering risk factors individually. 

In this investigation, Logistic Regression (LR), Decision Tree (DT), Random Forest (RF), XGBoost (XGB), and Gradient Boosting (GB) classifiers are used to predict whether posterior fusion surgery patients will experience a short or long LOS using the National Surgical Quality Improvement Database (NSQIP), a large repository of surgery case data supported by the American College of Surgeons (ACS) [8].

## 2. Methods

The ACS-NSQIP database was queried to select patients that underwent spine fusion surgery for ASD between 2006 and 2018. This cohort consisted of patients with current procedural terminology (CPT) codes of posterior (22800, 22802, and 22804) spinal fusions. A long LOS for posterior procedures was considered greater than or equal to 9 days. Both metrics are above the 75th percentile in their respective LOS distributions [6]. Patients with LOS equal to 8 days were excluded from the sample population. Hospitalizations shorter than 8 days for posterior spinal fusion were considered short LOS. A two-sided *t*-test was performed between key variables of the short and long LOS patient groups to determine statistically significant differences among both patient groups with a significance level set at 0.05, *a priori*.

Predictive variables included sex, race, American Society of Anesthesiologist’s (ASA) class, steroid use (Steroid), smoking history (Smoke), body mass index (BMI), surgeon Work Relative Value Units (Work RVU), age, and operating time (Op Time) as well pre-operative lab values: white blood cell count (WBC), creatinine levels (CREAT), platelet count (PLATE), hematocrit levels (HCT), blood urea nitrogen (BUN), sodium levels (SODM), and alkaline phosphatase (ALKPH). Work RVUs serve as a proxy for surgical invasiveness as they take into consideration factors, such as physician skill, effort, and time to perform the surgery. Patients that had undergone emergency surgery or had missing data in one or more missing predictive categorical values were excluded from the analysis. Due to the high proportion of missing preoperative laboratory values, a nearest neighbor imputation algorithm was applied to the missing laboratory values. Nearest neighbor imputations find correlations among patients with and without a value for a given variable. Based on these correlations, the nearest neighbor algorithm can be utilized to predict the missing value [9].

Patients were then randomly partitioned into two groups for the posterior procedural groups. The first group, consisting of 90% of the data, was designated as the training group. The second group, with the remaining patient data, formed the testing group. LR algorithms utilize a binomial logistic regression to predict prolonged LOS, while DT, RF, XGB, and GB algorithms use decision trees of various complexity to predict LOS. DT algorithms are less complex, and therefore, less thorough than RF algorithms. XGB and GB algorithms are the most complex algorithms, and therefore, they are thought to have the best predictive power. LR algorithms are less computationally intensive than DT, RF, XGB, and GB algorithms; however, this often comes at the expense of accuracy and predictability [10,11,12,13]. The five algorithms were used to predict patient LOS for the posterior patients with the Sci-Kit Learn package in Python (National Institute for Research in Computer Science and Automation, Rocquencourt, France) [14]. The algorithms’ predictive capacities were determined through analysis of the testing group. Prediction accuracy, area under the curve (AUC), and Brier scores were calculated to measure the strengths of the algorithms. Prediction accuracy is the proportion of patients correctly predicted as having a short or long LOS. AUC is a measure of how effective the algorithm is at distinguishing between patients who were and were not readmitted. The best AUC score is 1, indicating perfect distinctive capacity, while the worst AUC score is 0.5, indicating poor predictive accuracy [15,16,17]. Brier scores indicate how accurate the probabilities used to determine the predictions are, with a score close to 0 indicating high probabilistic predictive power, while a score closest to 1 indicates poor probabilistic predictive power [18].

## 3. Results

**Figure 1:**
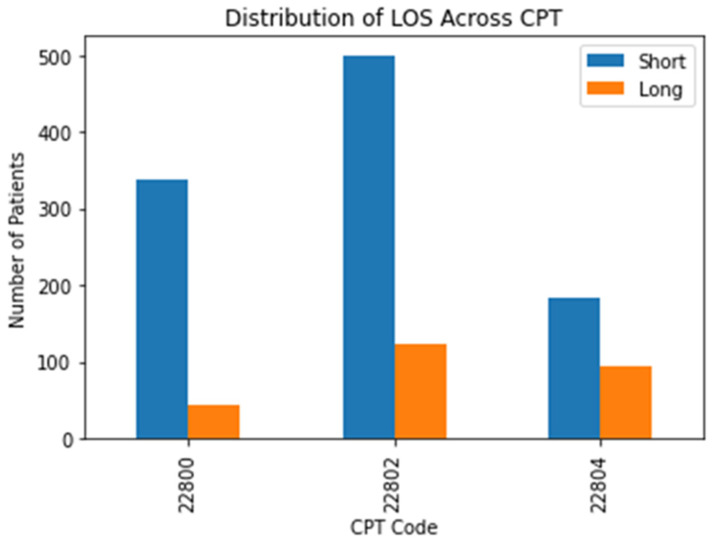
A breakdown of short and long LOS distribution by CPT code.

In total, 1907 patients that underwent posterior ASD surgery were examined. After removing missing values, 1281 patients were analyzed. The mean LOS for posterior ASD patients was 6.8 days. Among posterior cases, 262 patients had undergone a short LOS (20.5%). A comparative breakdown of short and long LOS cases by CPT code is displayed in Figure 1.

For the posterior fusion patients, the two-sided *t*-test results indicated that there was a statistically significant difference between short and long LOS among patients for the following variables: Caucasian, elevated ASA class, steroids, BMI, Work RVUs, age, operating time, HCT, BUN, and SODM. The full results of the two-tailed *t*-test of proportions and means are detailed in Table 1.

For posterior fusion patients, the five machine learning algorithms had AUC values of between 0.566 and 0.821 and prediction accuracies of between 68.4% and 83.1%. The Brier scores (Brier) for all five algorithms among posterior cases was close to 0, indicating a high prediction accuracy. The complete set of machine learning metrics are outlined in Table 2.

**Figure 2:**
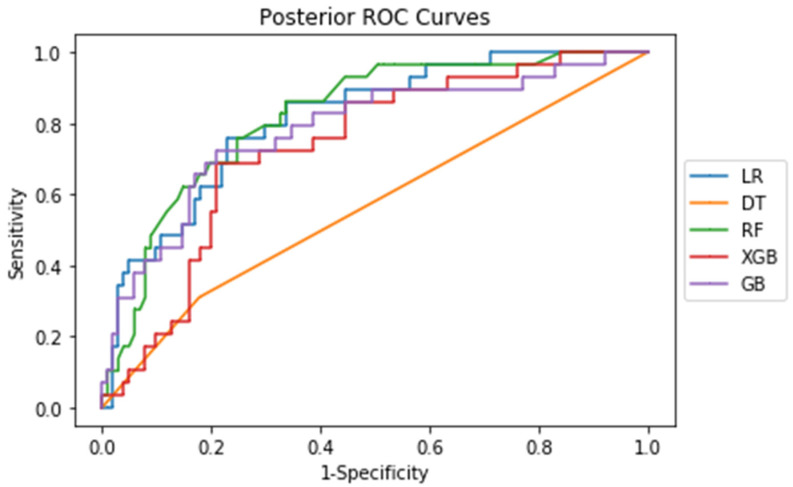
ROC curves of the five machine learning algorithms for posterior cases.

The corresponding ROC curves for each procedure show the tradeoff between the false positive and false negative rate (Figure 2). As seen from both ROC curves, sensitivity and 1-specificity are directly correlated, indicating that sensitivity and specificity are directly correlated.

**Figure 3:**
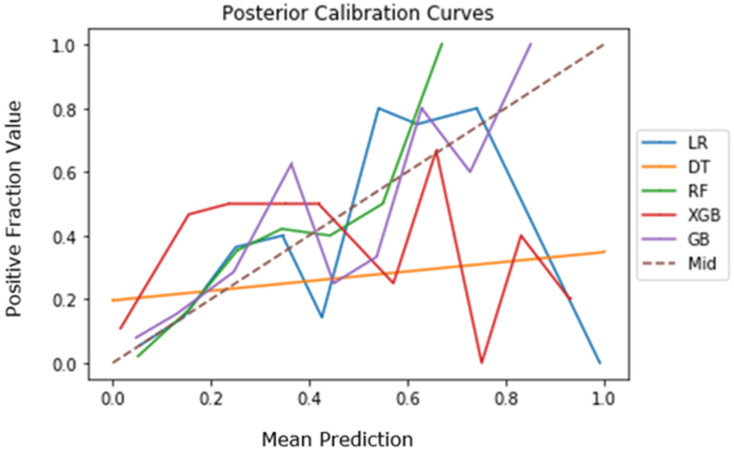
Calibration curves of the five machine learning algorithms for posterior cases.

The calibration curves for the five algorithms are depicted in Figure 3 for posterior cases. The XGB and GB algorithms align closest with the dotted midline (Mid), indicating strong accuracy relative to the other three algorithms for posterior cases [19,20].

**Figure 4:**
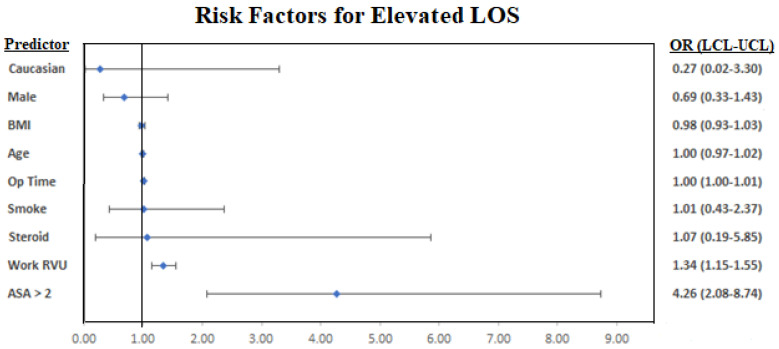
A forest plot visualization of the multivariable regression results. Lab values were removed from the forest plots due to near-zero confidence interval ranges and odds ratios close to 1.

The multivariable regression indicated that increased Work RVU, elevated ASA class, and longer operating times were linked to longer LOS for posterior cases (Table 3). A forest plot of the regression results is graphed in Figure 4.

**Figure 5:**
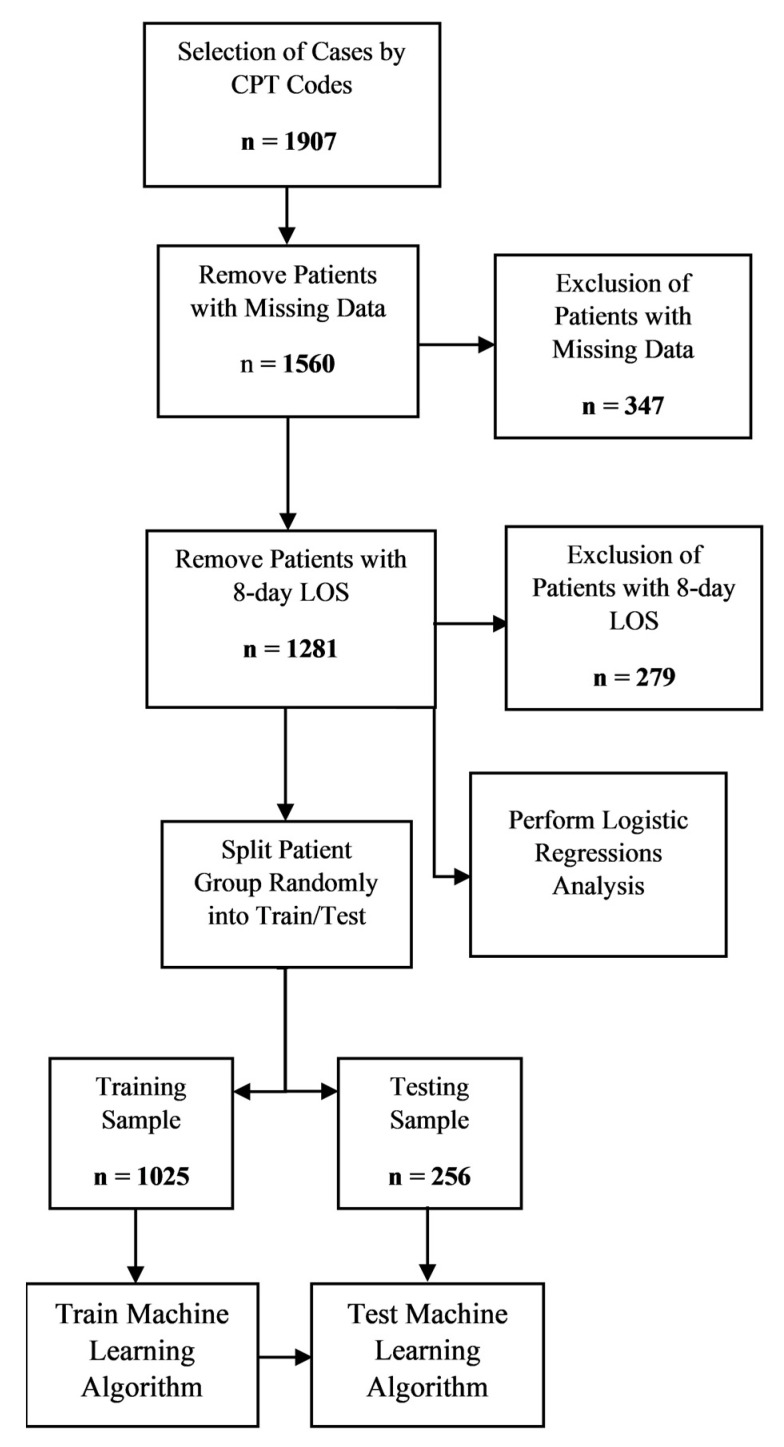
A flowchart visualization of the results.

A complete summary of the results is outlined in a flowchart in Figure 5.

## 4. Discussion

Prolonged LOS following ASD surgery has the potential to pose a substantial financial burden to both the patient and the healthcare system [4,19]. This is particularly seen in neurosurgery and orthopedic spine cases, where current research has found that an extended LOS was linked to higher complication rates and hospital costs among relevant cases. Ansari et al. found that prolonged LOS was associated with higher risks of readmission in neurosurgery patients; however, the paper concedes that this may have been due to underlying patient comorbidities rather than LOS itself [20]. Among lumbar fusion patients, extended LOS has been linked to increased risk of anemia requiring transfusion, altered mental status, pneumonia, readmission, and hardware complications requiring reoperation [21]. One previous study in neurosurgical patients revealed that a physical therapy consultation and discharge to a specialized nursing facility were both associated with a 2.4 day and 6.2 day longer LOS, respectively [22].

While many risk factors have been identified for increased LOS, no systematic model has been presented to determine whether a patient will experience a short or long LOS. This study not only found statistically significant differences in comorbidity and demographic related data between patients and their LOS, but more importantly, it demonstrated the efficacy of using tree-based machine learning techniques to predict short vs. long LOS. The implications of these results are significant because they contribute to a more comprehensive understanding of a patient’s individual risk profile and may allow for more appropriate resource allocation and accurate discharge planning practices. The variables used in the machine learning algorithm have been used to formulate a user interface webpage link for LOS prediction after ASD (https://asdlos.herokuapp.com/).

Four of the five algorithms (LR, RF, XGB, and GB) tested in this study had AUC values greater than 0.7 and were, thus, highly effective in predicting and distinguishing between the test cases [23]. The results of this study compare favorably against similar publications exploring the implementation of machine learning in both spine and other orthopedic subspecialties. Prior studies using machine learning to predict intraoperative blood loss, prolonged LOS, patient reported outcome measures, and discharge disposition in the fields have utilized similar tree-based approaches or neural networks with similar or inferior results [24,25,26,27,28,29]. The results of this paper provide further evidence that machine learning has much utility in orthopedic surgery, as the current study builds upon existing literature using machine learning in spinal surgery. Moreover, Kobayashi et al. found that elevated ASA Class and longer operating times were associated with increased LOS after posterior spine fusion, of which the results of this study concur with [30]. Adogwa et al. identified surgeon practice style and preference as risk factors for extended LOS, which aligns with our study’s findings that surgeon Work RVU and operating time were associated with increased LOS [31]. However, operating time cannot be solely attributed to surgeon style or preference, as there may be patient comorbidities that lead to an increased operative time [32].

The Risk Assessment Prediction Tool (RAPT) is a validated six-question survey designed to predict patient discharge disposition following total joint arthroplasty. It helps both patients and physicians better understand barriers to discharge and aids in the shared decision-making process to streamline the discharge process [33]. Similarly, the results of this study could be extrapolated as a foundation for the development of a similar tool. It would be tremendously helpful for patient and physician alike to be able to quantify a patient’s risks prior to surgery. This not only allows for a more appropriate setting of expectations, but also enables the entire patient care team to devise an individualized care plan aimed at maximizing patient safety and satisfaction while simultaneously reducing length of stay.

Despite the strength of the machine learning algorithms presented, the study does have some potential limitations. The NSQIP dataset is a national database, and the results of studies from the database may not be clinically relevant to individual hospitals or medical institutions. Furthermore, for the algorithms to be most clinically useful, they must be based on a sample that is truly reflective of the population of patients undergoing spine fusion surgery for ASD treatment. However, the NSQIP database is overrepresented with patients from large teaching hospitals and underrepresented with patients from smaller community hospitals. Thus, the algorithms presented here are based on somewhat skewed data [34]. Moreover, due to the inherent limitations of CPT coding, we were unable to distinguish between cervical, thoracic, and lumbar spinal constructs in the patient population. Finally, the number of patients undergoing spine fusion surgery for ASD treatment is characteristically low, thus reducing the power of any inferential statistical procedure, such as machine learning [35].

## 5. Conclusions

The results of this investigation indicate that machine learning algorithms, specifically tree-based, are effective in predicting the LOS duration for patients undergoing spinal fusion surgery to treat ASD. The clinical implications of these algorithms can be immense, as patients and their providers can be empowered with a way to predict the expected LOS after surgery. With this information in hand, specific measures can be taken to reduce LOS and, consequently, reduce expenses for both patients and hospitals alike. More research is warranted to determine the overall effectiveness of these algorithms relative to hospital-specific cases.

## Figures and Tables

**Table 1:** A demographics table of patients with short and long LOS for posterior spine fusion cases.

	Short LOS	Long LOS	*p*-Value
% Male	360 (34.88%)	85 (32.44%)	0.46
Caucasian	794 (76.94%)	156 (59.54%)	<0.0001
ASA > 2	530 (51.36%)	210 (80.15%)	<0.0001
Steroid	44 (4.26%)	20 (7.63%)	0.02
Smoke	159 (15.41%)	42 (16.03%)	0.8
BMI	27.3	28.5	0.01
Work RVU	28.9	32	<0.0001
Age (years)	51.3	57.5	<0.0001
Operation Time (minutes)	321	440.5	<0.0001
WBC	7.1	7.4	0.18
CREAT	0.9	0.9	0.3
PLATE	252.5	249	0.49
HCT	40.3	38.9	<0.0001
BUN	15.3	16.3	0.03
SODM	139.2	138.5	<0.0001
ALKPH	83.9	89.6	0.06
LOS	4.5	15.3	<0.0001
Total	1032	262	

**Table 2:** The results of the machine learning analysis of the five machine learning algorithms for the posterior cases.

	AUC	Prediction Accuracy (%)	Brier
LR	0.814	83.1%	0.13
DT	0.566	68.4%	0.29
RF	0.821	78.5%	0.14
XGB	0.736	73.1%	0.20
GB	0.782	80.8%	0.14

**Table 3:** Results of the multivariable regression analysis for posterior cases.

	Posterior			
	Odds Ratio	Lower	Upper	*p*-Value
BMI	0.98	0.93	1.03	0.49
Work RVU	1.34	1.15	1.55	<0.001
Age	1.00	0.97	1.02	0.71
Male	0.69	0.33	1.43	0.32
Caucasian	0.27	0.02	3.30	0.31
ASA > 2	4.26	2.08	8.74	<0.001
Steroid	1.07	0.19	5.85	0.94
Smoke	1.01	0.43	2.37	0.98
Op Time	1.00	1.00	1.01	<0.001
WBC	1.02	0.86	1.22	0.82
CREAT	0.42	0.08	2.12	0.29
PLATE	1.00	1.00	1.01	0.29
HCT	0.95	0.88	1.03	0.20
BUN	1.01	0.95	1.08	0.74
SODM	1.04	0.93	1.17	0.50
ALKPH	1.00	0.99	1.01	0.84

## Data Availability

This study used the American College of Surgeon’s NSQIP dataset. https://www.facs.org/quality-programs/acs-nsqip (accessed on 22 January 2021).
